# Evaluation of whole-genome enrichment and sequencing of *T. pallidum* from FFPE samples after 75 years

**DOI:** 10.1016/j.isci.2023.108651

**Published:** 2023-12-08

**Authors:** Vincent Zvenigorosky, Angéla Gonzalez, Gilles Veith, Tricia Close-Koenig, Catherine Cannet, Jean-Luc Fausser, Alexandre Wenger, Laurence Toutous-Trellu, Christine Keyser, Christian Bonah

**Affiliations:** 1 SAGE Laboratory, CNRS UMR 7363, Strasbourg, France; 2 Strasbourg Institute of Legal Medicine, Strasbourg, France; 3 Interfaculty Centre for Bioethics and Medical Humanities, University of Geneva, Geneva, Switzerland; 4 University Hospitals of Geneva, HUG, Geneva, Switzerland; 5 BABEL Laboratory, CNRS UMR 8045, Paris, France

**Keywords:** Medical microbiology, Medical biotechnology

## Abstract

The recent developments in genomic sequencing have permitted the publication of many new complete genome sequences of *Treponema pallidum pallidum*, the bacterium responsible for syphilis, which has led to a new understanding of its phylogeny and diversity. However, few archived samples are available, because of the degradability of the bacterium and the difficulties in preservation.

We present a complete genome obtained from a Formalin-Fixed Paraffin-Embedded (FFPE) organ sample from 1947, kept at the Strasbourg Faculty of Medicine. This is the preliminary, proof-of concept study of this collection/biobank of more than 1.5 million FFPE samples and the evaluation of the feasibility of genomic analyses.

We demonstrate here that even degraded DNA from fragile bacteria can be recovered from 75-year-old FFPE samples and therefore propose that such collections as this one can function as sources of biological material for genetic studies of pathogens, cancer, or even the historical human population itself.

## Highlights

• FFPE samples can still be used for genomic studies 75 years after embedding
• Whole-genome enrichment can be successful even with fragile degraded bacteria
• A new avenue of research into symptom-strain correlation in diseases like syphilis
• Archived FFPE samples and medical records constitute potential biobanks of samples

## Graphical abstract

**Figure:**
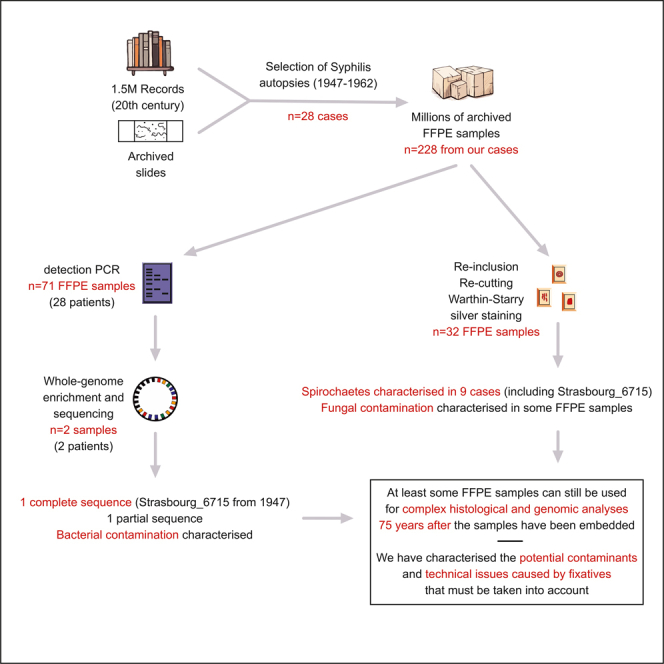

## Introduction

The Institute of Pathological Anatomy of the Faculty of Medicine of the University of Strasbourg has preserved around 1.5 million formalin fixed paraffin embedded (FFPE) samples, used for diagnoses related to clinical practice and autopsies over the twentieth century. This exceptional collection is associated with thousands of medical, autopsy, histology, and other records, making the ensemble a unique historical and biological resource. Although the usability of FFPE as a source of genomic DNA has been demonstrated,^1^^,^^2^ this collection is exceptionally old, and significant degradation was expected.^3^ FFPE are however one of the only types of medical samples that remain a source of DNA decades after collection^4^^,^^5^^,^^6^ and the only one that can be preserved at room temperature.

Our research focuses on syphilis because this sexually transmitted disease is both of historical interest and an ongoing public health concern.^7^^,^^8^ The number of genetic studies of the pathogenic spirochaete bacterium responsible for syphilis in humans, *Treponema pallidum pallidum* (thereafter referred to as TPA), has increased in recent years,^9^ motivated by the resurgence/persistence of the disease in the western hemisphere. TPA is an obligate parasite,^10^ and therefore could not be grown in the lab until very recently, and not without difficulty,^11^ a fact that also precluded the use of many genomic analysis techniques, especially before the advent of Next Generation Sequencing (NGS) technologies.^9^ Lineage conservation in rabbits carries specific drawbacks, such as the appearance of new genomic variants that were not present in infected patients,^12^ but a larger drawback is the long-term preservation of frozen samples, which has historically been rare because it was unjustified before the advent of molecular genetics.

The diagnosis of syphilis improved over time,^13^ including the recent sequencing of a complete genome from an FFPE sample.^14^ Over the history of syphilis, however, detection methods have been somewhat unreliable^15^^,^^16^^,^^17^^,^^18^ and the diagnosis of syphilis singularly complex.^19^^,^^20^ Syphilis was also historically over-diagnosed for a variety of cultural or social reasons: the prevalence of the pathology in certain groups led to different disorders and rashes being attributed to syphilis without adequate testing. After the discovery of penicillin and the dramatic improvement of treatment, over-diagnosis was also maintained by the perceived innocuousness (partially justified) of prescribing antibiotics even in the absence of positive evidence of the disease.^21^^,^^22^

Three subspecies have been described within the *Treponema pallidum* species, which cause yaws and bejel (two tropical diseases) and syphilis, caused by TPA. The more recent evolution and diversification of this bacterium is due to five factors: (1) it is a persistent pandemic, (2) it has been left untreated in some regions or groups, (3) it has been historically treated with ineffective methods, (4) it has sometimes been treated with (or exposed to) macrolide antibiotics instead of derivatives of penicillin and (5) it coexists with the two aforementioned diseases in tropical regions and other sexually transmitted infections around the world. Consequently, aside from sequencing for direct medical applications (namely vaccine development) and simpler methods of detection, population genetics studies of TPA focus on three aspects of the genome: (1) the assignment to the major clusters (namely strains Nichols and SS14^23^) and their evolution, (2) the evolution of the variant (point mutation) associated with macrolide resistance^24^ and (3) the interactions between the three known subspecies.^25^ We therefore performed analyses with the aim of detecting the bacterium, characterizing the strain and determining whether a sample contained macrolide-resistant TPA.

## Results

### Histological positives

**Figure 1:**
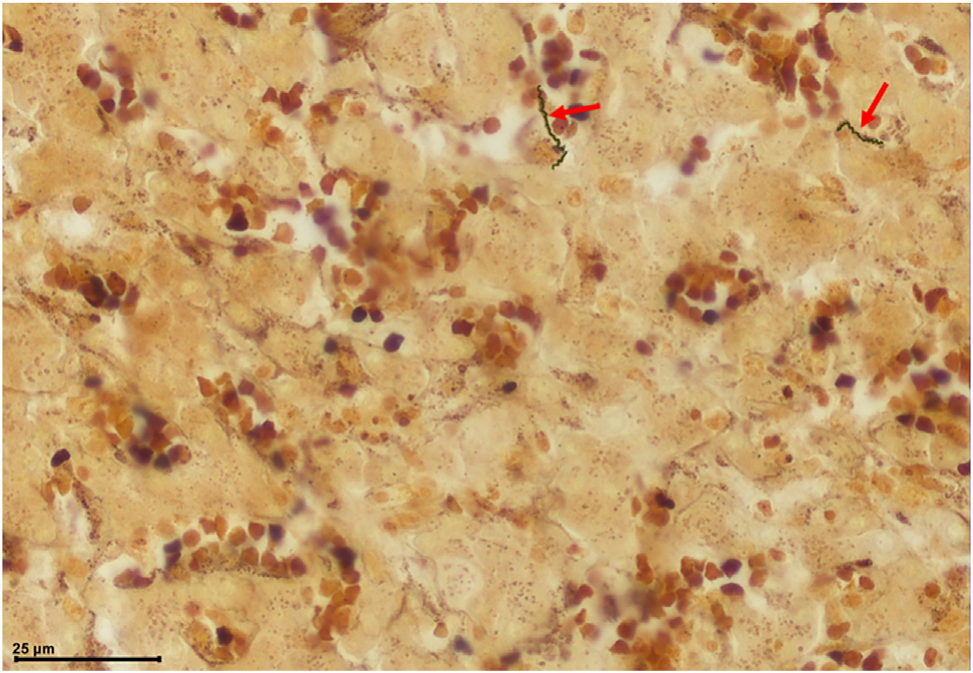
Histological characterization of case 6715 Liver sample from a congenital syphilis case in 1947, fixed in formalin; red arrows indicate spirochaete bacteria.

Of the 32 re-included blocks tested using the Warthin-Starry staining method,^26^ 9 showed the presence of spirochaete bacteria [Table 1], surrounded by inflammatory infiltrate, as observed under a microscope [Figure 1 and Supplementary Materials and Methods (Observed Spirochaete)]. The presence of fungi was noted in some of the newly made slides [Supplementary Materials and Methods], indicating that the paraffin blocks themselves were contaminated.

**Table 1:** Summary of results

Case number	WS	PCR	CE sequence	12 SNP multiplex	Genome
6709	2	4	4	12 (SS14)	32.25%
6710	(−)	nt	nt	nt	nt
6715	1	2	1	11 (SS14)	99.66%
7709	nt	(−)	nt	nt	nt
7767	1	3	2	2	nt
7773	nt	(−)	nt	2	nt
7781	(−)	(−)	nt	3 (SS14)	nt
7828	nt	1	(−)	nt	nt
7840	(−)	1	1	5	nt
8237	2	nt	nt	nt	nt
8543	nt	(−)	nt	3 (Nichols)	nt
9218	(−)	(−)	nt	nt	nt
9754	nt	1	(−)	3 (Nichols)	nt
9904	nt	2	(−)	nt	nt
9905	nt	(−)	nt	2	nt
10176	1	(−)	nt	nt	nt
10441	(−)	1	1	4	nt
10951	nt	1	(−)	3	nt
12261	nt	(−)	nt	2	nt
12323	(−)	nt	nt	nt	nt
12435	1	(−)	nt	nt	nt
13173	nt	(−)	nt	nt	nt
13491	nt	(−)	nt	nt	nt
13503	nt	(−)	nt	nt	nt
13577	1	1	1	3 (Nichols)	nt
14939	nt	(−)	nt	nt	nt
15092	nt	(−)	nt	3 (Nichols)	nt
15421	nt	1	(−)	3 (Nichols)	nt
6721 (−)	nt	(−)	nt	nt	nt
6742 (−)	nt	(−)	nt	nt	nt
7763 (−)	nt	(−)	nt	nt	nt
Positive control	nt	4	4	12 (Nichols)	nt

WS: Warthin-Starry staining; PCR: simple detection; CE: Capillary Electrophoresis; nt: not tested; (−): negative; we did not obtain sequences that did not correspond to TPA using CE.

### PCR positives

The targeted fragment was detected [Table 1] using capillary electrophoresis in 18 out of the 71 FFPE samples tested (25%), corresponding to 11 of 25 patients (44%) and this was confirmed using capillary sequencing in 10 out of 18 cases (determination of the sequence failed in 8 cases). No results were obtained from three of the four cases in which paraffin blocks had undergone fixation using Bouin. However, the one positive obtained after Bouin fixation (case 6709) corresponded to the most successful of all samples, with four separate PCR positives.

### 12 SNP multiplex

Only cases 6709 and 6715 gave satisfactory results (11 and 12 SNP respectively, less than 5 for all others) and both could be assigned to the SS14 strain [Table 1]. These samples were therefore selected for targeted enrichment and whole-genome sequencing.

### Complete genomes

Enrichment and Illumina MiSeq sequencing of the two selected high-quality samples (6709 and 6715) produced around 7 million reads. We mapped sequenced reads on the SS14 TPA reference genome (access number GenBank: CP004011.1), but the majority of reads did not map to that reference. There were 99.64% of unmapped reads for sample 6709 (4,217,078 out of 4,232,247, or 15,169 mapped reads) and 81.89% of unmapped reads for sample 6715 (1,252,682 out of 1,529,671, or 276,989 mapped reads). This was the consequence of unusually high coverage (>10000x) for 50 of the designed probes, almost exclusively covering the 23S rRNA gene, which is present in two copies.

We used the Kraken program to characterize unmapped reads and we detected contamination from both bacterial and human DNA. The most common bacterial contaminants belonged to bacteria of the Burkholderia and Paraburkholderia genera and *Cutibacterium acnes* (all around 10% of the reads in sample 6709 but less than 1% in sample 6715), as well as *Schlegelella aquatica* (around 1.1% in sample 6709). Out of the 50 over-represented regions, 24 corresponded specifically to Burkholderia and explained the majority of unmapped reads. Despite excluding the human genome in our design, human sequences make up 36.2% of contaminants in sample 6709 and 93.6% in sample 6715. These did not, however, hamper the analysis in any detectable way. We could not recover enough DNA from the same FFPE samples to repeat the analysis using another panel excluding the now known contaminants.

The final consensus sequence for sample 6709 only covered 32.25% of the bacterial genome at a depth superior to 1x (the average depth was 0.71x). It was therefore not included in phylogenetic analyses. Sample 6715 yielded a near-complete sequence, with 99.66% of the genome covered over 1x and an average sequencing depth of 13.99x. The sequenced genome of the bacterium for case 6715 was deposited into the NCBI database (accession number CP115658) and both Fasta-format files are provided in Supplementary Materials (Supplementary Files SF1 and SF2). The variants associated with macrolide resistance were not found in either genome and the reliability of this result is supported by the good coverage of these regions for both samples.

### Phylogeny

**Figure 2:**
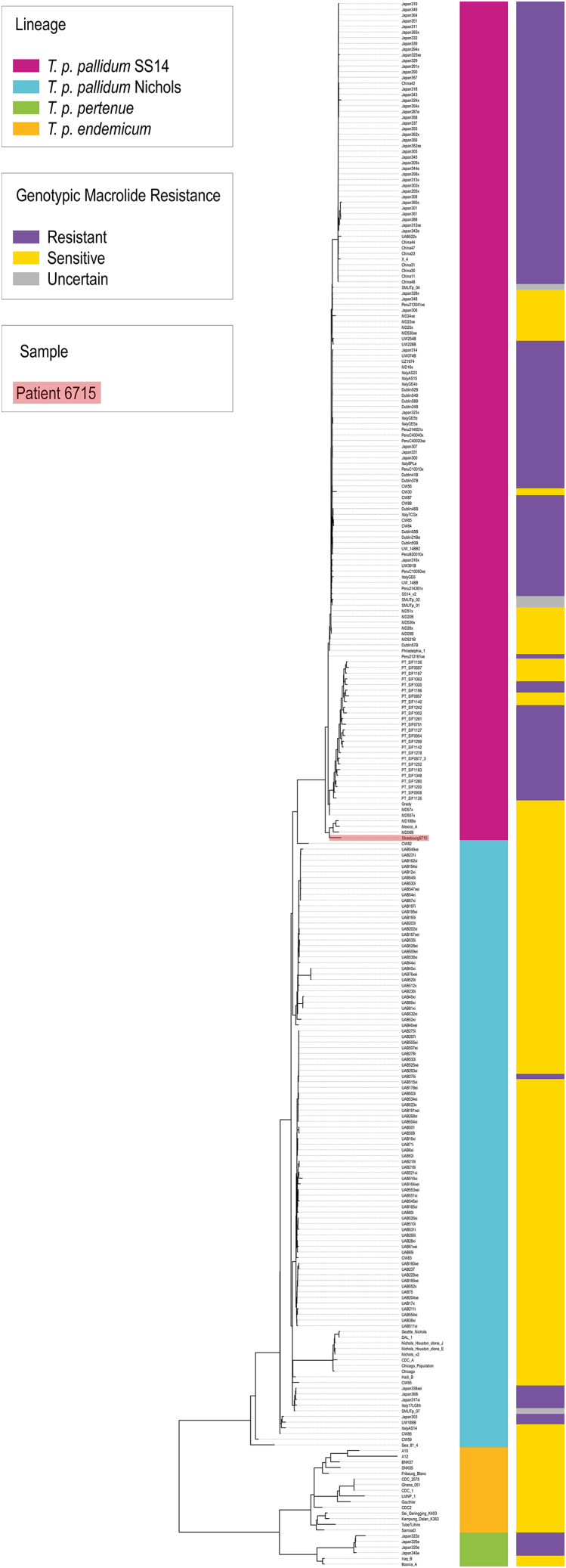
Phylogeny of Strasbourg 6715

Our results [Figure 2] place the genome of Treponema recovered from case 6715 (Strasbourg_6715) in the SS14 cluster (as also indicated by the multiplex). More specifically, the genomes closest to the Strasbourg_6715 sequence are Mexico-A (Mexico, 1953^25^^,^^27^), MD18Be (USA, 1998^28^), and MD06B (USA, 2002^28^). We computed pairwise differences (single indels and substitutions) between Strasbourg_6715, the aforementioned sequences, the SS14 reference, the Nichols reference and CW82, the Nichols sequence closest to the SS14 cluster. We find that Strasbourg_6715 is equidistant from Mexico-A, MD18Be and MD06B on one side (182 pairwise differences on average) and the SS14 reference on the other (160 pairwise differences). The strains in the Mexico-A cluster are closer to one another (<87 differences) than they are to either Strasbourg_6715 or SS14 (159–209 and 99 to 127 differences, respectively).

These pairwise differences are distributed throughout the genome [Figure S7] but a few regions of interest show higher variability between Strasbourg_6715, the Mexico-A cluster, SS14 and CW82: the two 23S ribosomal RNA regions, TprI, TprJ, and TprK.

## Discussion

### Successful histological and genetic detection of TPA

Previous studies have demonstrated the possibility of extracting DNA from FFPE samples stored for up to 40 years.^5^ The present work shows the possibility of confirming past diagnoses from FFPE samples older than 70 years, even when storage conditions are sub-optimal. We have detected the presence of DNA endogenous to the sample (human or bacterial) in most paraffin blocks and confirmed half (14/28) of the past syphilis diagnoses using either histological staining or PCR. Given the complexity of the staining method and the scarcity of the bacterium inside the tissue after the removal of the inflammation area, this is a very satisfactory result. This also opens the possibility of analyzing other markers, such as methylations, which have so far been characterized in 30-year-old FFPE samples^4^ or RNA sequences, obtained after 20 years.^29^

These results also exemplify the difficulties inherent in working with archived clinical samples. All paraffin blocks produced without standardization or automation had to be re-embedded in cassettes (up to four different cassettes for the larger ones, increasing the number of slides to be examined). Only after delineation of regions of interest for genetic analyses on H&E-stained slides, re-cutting, and re-embedding, could the blocks be re-stained. Moreover, the Warthin-Starry method itself is a time-consuming procedure without automation and calls for very specific expertise, especially on fragile archived samples.

The contamination of blocks and slides by fungi resulting from temperature variation and humidity did not affect the stains but highlighted the necessity for such invaluable material to be stored in an appropriate environment.

### Ideal samples for DNA analysis

There does not appear to be a correlation between the dates of collection and the efficacy of genetic analyses. Positive samples range from 1947 to 1962 and negative samples range from 1950 to 1962. The age of the patients at death is not evidently correlated but the most successful samples corresponded to the younger patients: four positives for case 6709 (six weeks old), two positives for case 6715 (nine days old), three positives for case 7767 (43 years old) and two positives for case 9904 (39 years old). There is also no detectable correlation between the number of paraffin blocks/samples analyzed and the number of positives. Instead, there is great variability between cases and blocks corresponding to any single case.

Although there is no clear correlation between the age of individuals and the quality of the DNA extracted, the analysis of histological records confirms one of the likely explanations for the success or failure of individual analyses: fixation time in formalin.^30^ Our records do not indicate the delay between sample collection and histological analysis *per se*, but they mention the date of autopsy and often the date at which the report was finished and signed. From this information, we can infer that after the autopsy of younger patients, histological analyses were usually performed sooner after death. This could be explained by the necessity of confirming a diagnosis after an untimely death and is especially true in cases 6709 and 6715, two very young infants, for which histological analyses were possibly intended to confirm the serological status of their respective mothers (it is unclear whether the detection of syphilis would have been accurate enough for these women at the time). Further study of the records could allow for better estimations of the duration of each preparatory step and help select the FFPE samples most likely to yield high-quality DNA.

### Phylogeny

The most notable cluster related to sample 6715 composed of three genomes from samples collected in 1953, 1998, and 2002, in Mexico and the USA. This group has been described as the Mexico-A cluster^25^^,^^31^ or the SS14-like cluster^32^ and is an outlier compared to other SS14 sub-strains.^33^ Genome Strasbourg_6715 is as distant from this cluster as it is from other SS14, which places it closer the divergence point between those two branches and makes it another outlier within the SS14 cluster. Based on these 7 accurately dated sequences, we can estimate a mutation rate of 0.58 per genome per year.

### Perspectives

The lack of knowledge of potential contaminants led to the design of an enrichment panel that favored the contaminant over the organism of interest in certain genomic regions. While these results are somewhat specific to our collection, they certainly warrant caution in all cases and will permit the design of better-adapted panels for the sequencing of Treponema genomes or markers of other diseases in the future. Burkholderia bacteria in our FFPE samples likely originate from the direct environment of the autopsy, collection area or storage area, and *Cutibacterium acnes* could have been introduced by the examiners, the lack of proper sterilization of the instruments, the reuse of instruments or any manipulation of the bodies.

Treponema bacteria are especially fragile and PCR detection at different stages of the disease can be ineffective, even in living tissue or biological fluids. The fact that the present study was able to successfully sequence a full Treponema genome from 75-year-old FFPE material is an indication of the possibilities regarding other, more durable bacteria, as well as genetic markers of cancer, viral infection, or other infectious or non-infectious diseases.

It is also evident that the recovery of human DNA from the same samples would present far fewer difficulties and could be of many uses. This collection represents a cross-section of the entire Alsatian population from 1947 to the 2000s and includes a great number of different diseases and conditions.

### Conclusion

We have shown that genetic material can be recovered from most FFPE samples from the 1940s onward and that bacterial genetic material can also be recovered if the histological treatment of the samples falls within certain parameters. In some cases, it is even possible to recover enough material to succeed in sequencing whole bacterial genomes. Complex histological staining can be applied to the material and spirochaete bacteria can be observed after 75 years. This study should serve to highlight the necessity of preserving knowledge of techniques now considered obsolete or unnecessarily complex, without which the analysis of these archived samples would have been more difficult.

Collections of archived FFPE samples associated with extensive technical and medical records are not commonplace, and the present study demonstrates that their value exceeds their historical significance. Such collections are potential sources of human, bacterial, viral, parasite, or tumor DNA (or RNA from more recent FFPE samples). Retrospective studies could be performed using hundreds or thousands of confirmed cases of any given pathology. It is, therefore, necessary to ensure the preservation of such records, by improving storage conditions, digitizing written records, transcribing the information into a usable format, and standardizing genetic and histological protocols.

The complete genome presented here represents the oldest TPA sequence obtained directly from a clinical sample and its analysis reveals the presence of the SS14-like strain earlier than previously described (and in Europe). It therefore appears essential to proceed with the analysis of all archived syphilis samples available in this collection or other collections, to better understand the phylogenetic history of the bacterium.

### Limitations of the study

Our research was limited to retrieving syphilis cases associated with postmortem samples. This is because, unlike other pathologies that might lead to biopsies and sampling in living patients, tissue samples are not taken from living patients in suspected cases of syphilis. Diagnosis is based on serological samples. Samples from living patients, which might provide valuable insights, are generally not preserved.

The complete genome we have obtained (Strasbourg_6715) was retrieved from a sample corresponding to a case of congenital syphilis, in which the treponemal burden is higher than in other cases. Further analyses are therefore necessary to confirm that PCR positives obtained from adult cases could also lead to the successful sequencing of complete genomes.

The impact of fixatives used in sample preservation also remains largely unexplored. Although we initially assumed that the use of Bouin fixative could make subsequent analyses unfeasible, our study found that this was not the case.

The presence of fungi and other contaminants highlighted the complex degradation conditions of the FFPE. These samples were not preserved in well-controlled conditions (i.e., temperature, humidity, dust). This is crucial information for similar archives yet to be mobilized, because it confirms that conditions within such environments can significantly affect the integrity and analytical outcomes of the samples.

## STAR★Methods

### Key resources table

**Table:** 

REAGENT or RESOURCE	SOURCE	IDENTIFIER
**Biological samples**		

Archived FFPE samples 1947 to 1970	This paper	N/A
Control sample – chancre swab HUG	This paper	N/A
Rabbit testicle tissue containing Treponema pallidum	Sigma-Aldrich, Germany	ref TTR011-25EA

**Deposited data**		

Strasbourg_6715 complete sequence	This paper	NCBI: CP115658

**Oligonucleotides**		

Detection PCR primers (Supplementary Methods)	This paper	N/A
Multiplex primers (Supplementary Methods)	This paper	N/A
Custom panel of 14216 80bp probes covering 99.85% of the TPA genome (NCBI: CP004011.1) with no tiling	TWIST Bioscience	N/A

**Software and algorithms**		

EAGER pipeline v2.4.5	Peltzer et al.^34^	N/A
FastQC V0.11.9	Andrews^35^	https://www.bioinformatics.babraham.ac.uk/projects/fastqc/
AdapterRemoval V2.3.2	Schubert et al.^36^	N/A
BWA v0.7.17	Li et al.^37^	N/A
markduplicates v2.26.0	^38^	http://broadinstitute.github.io/picard/
Samtools v1.16.1	^39^	https://github.com/samtools/samtools/releases/tag/1.16.1
MAFFT v7.505	Katoh et al.^40^	N/A
iqtree v2.0.3	Minh et al.^41^	N/A
TempEst v1.5.3	Rambaut et al.^42^	N/A
ape package for R	Paradis et al.^43^	N/A
R	R Core Team^44^	https://www.R-project.org/
Kraken 2.1.3	Wood et al.^45^	N/A
KrakenUniq	Breiweiser et al.^46^	N/A

### Resource availability

#### Lead contact

• Further information and requests for resources and reagents should be directed to and will be fulfilled by the lead contact, Vincent Zvenigorosky (vincent.zvenigorosky@gmail.com).

#### Materials availability

• The custom panel of 14216 80bp probes covering 99.85% of the TPA genome with no tiling that was designed by the TWIST Bioscience supplier is maintained by them.
• The Detection PCR and Multiplex primers are reported in Supplementary Materials.

#### Data and code availability

• The sequence for Strasbourg_6715 was deposited in the publicly accessible GenBank database under accession number CP115658. The partial sequence for Strasbourg_6709 is provided as Supplementary File SF1. All other data used in the analysis is included in the Supplementary Tables.
• This paper does not report original code.
• Any additional information required to reanalyse the data reported in this paper is available from the lead contact upon request.

### Experimental model and study participant details

• One chancre swab was provided by our partners at the Hôpitaux Universitaires de Genève and used as a positive control in all analyses. Written informed consent was obtained from the participating patient and anonymity was preserved throughout (no identifiers were associated at any point).

#### Ethics committee approval

This study received approval from the Comité d’Ethique des Facultés de Médecine, d'Odontologie, de Pharmacie, des Ecoles d’Infirmières, de Kinésithérapie, de Maïeutique et des Hôpitaux de Strasbourg (reference number: CE-2022-124).

### Method details

All experiments and methods were performed in accordance with relevant guidelines and regulations.

#### Planned progression of analyses for the proof-of-concept

Our objective was to submit as many samples as could be retrieved from the archived materials at the Institute of Pathological Anatomy of the Strasbourg Faculty of Medicine to histological and genetic analyses. We planned an analytic progression from the most likely to succeed to the least likely. Histological analyses were limited by the age of the material, and we therefore focused on direct microscopic observation of unstained slides, stained slides and Warthin-Starry silver staining [Supplementary Materials and Methods]. Genetic analyses were carried out as follows: mitochondrial HV1 region amplification (checking for the presence of DNA, data not shown), detection PCR for TPA, CE sequencing of the same fragments, 12 SNP multiplex (to characterize strains) and finally whole-genome targeted enrichment and sequencing. At each stage, we selected only the samples we believed could yield results in the next in order to conserve DNA extracts for future analyses. The details of the molecular methods are given in Supplementary Materials and Methods, except for the targeted enrichment and NGS sequencing which is presented below.

#### Selection of the cases in this proof-of-concept study

Histological and autopsy records from January 1947 to July 1962 were studied and any mention of Syphilis was noted, even in cases where this diagnosis was eventually rejected by the histopathologist. We selected 28 cases of syphilis [Table 1], which were associated with a total of 228 FFPE organ samples [Table S1] and the corresponding unstained and stained slides. We also selected 3 control cases with different diagnoses. The scanning and processing of the records is ongoing, but we estimate the total number of autopsies performed at the Institute on patients that died of syphilis or with syphilis for the 1947-1970 period to be around 80. With more than 28000 autopsies performed during this period, it should be noted that the prevalence of syphilis in the collection is likely lower than it would have been in the living population. Some cases would not have been fatal and/or detected at autopsy.

#### Historical techniques

Formalin-Fixed Paraffin Embedded organ samples from 1947 to 1962 were stored at the Institute of Pathological Anatomy of the Strasbourg Faculty of Medicine in a room subjected to important variations of temperature and hygrometry. Before the 1970s (all the samples presented in this study), histological technique was exclusively manual, and inclusions were performed as follows:

• The fixatives used were formalin and Bouin solution (a fixative composed of picric acid, formaldehyde, and acetic acid)
• Staining was either H&E (Haematoxylin and Eosin), HES (Haematoxylin, Eosin, and Saffron) or Fuchseline (resorcinol/fuchsine)
• Sampled and cut organs were set in capsules of varying sizes (1·0 cm x 0·5 cm to 3·0 cm x 6·0 cm)
• Dehydration, clearing and hot paraffin infiltration were performed in tanks and neither the time spent in each solution, nor the temperature of the paraffin are known or recorded
• The FFPE blocks were made using Leuckart embedding irons of varying sizes (1·5 x 1·5 x 0·7 cm to 6·0 x 4·5 x 0·7 cm) [Figure S1]
• Technicians engraved the patient number on one of the sides of each block using a steel point. All blocks from the same patient share a unique number.
• Blocks were cut using large razorblades fitted into a manual microtome

#### Modern treatment of the material

In order to identify each organ separately, we added a unique number to each block and the corresponding slides. Inconveniently sized blocks were re-cut into standard cassettes [Figure S2]. We then proceeded with Warthin-Starry silver staining (010270, Diapath, MM France). Positive control slides were paraffin sections of rabbit testicle tissue containing *Treponema pallidum* (ref TTR011-25EA, Sigma-Aldrich, Germany).

Warthin-Starry is a silver stain based on an argyrophilic reaction.^26^ Spirochaetes are argyrophilic, that is to say they can be impregnated with silver, but a reducing agent is needed to reduce the silver to visible metallic silver. Slide tissue sections are first immersed in an acidified aqueous solution of silver nitrate (optimum pH 3·5-4·0) then immersed in a reducing solution that typically contains hydroquinone, gelatine, and a lower concentration of silver nitrate. The procedure causes the spirochetes to be stained dark brown to black which contrasts with a paler yellow background (see Observed Spirochaete and contaminants section below).

#### Selection of the samples

We performed analyses on the FFPE samples based on preliminary evaluation by the histologist. Our criteria for exclusion were the following: no observable inflammation, decalcification, negative comments about the technique by the original pathologist (usually excessive fixation time, “cooked” samples), poor fixation, poor dehydration and/or degradation of the block during storage. The specific characteristics of some samples made them suitable for one analysis or the other but not both, especially the location of the inflammation, the size of the sampled organ and the type of fixative used.

We selected 71 FFPE blocks from the 25 cases of syphilis for genetic analyses, as well as 3 negative controls [Table 1] and 32 FFPE blocks from the 25 cases of syphilis for re-inclusion, cutting, and staining.

#### Extraction of DNA from archived FFPE samples

The surface was cleaned with sterile compresses soaked in ultra-pure water and a thin layer of paraffin was removed with a scalpel. Tissue samples were taken from the entire depth of the embedded tissue with a second sterile scalpel, focusing on the inflammation area as determined by the observation of the stained slides. The quantity recovered was sufficient to perform several extractions from one block.

The protocol steps followed were those of the GeneRead DNA FFPE kit (QIAGEN) specifically dedicated to DNA extractions from paraffin-embedded tissues. This kit was then replaced by the next-generation QIAamp DNA FFPE Advanced Kit, Cat.no. 56704 QIAamp DNA FFPE Advanced UNG. The different steps of the DNA extraction protocol were therefore performed as recommended by the supplier QIAGEN.

The final elution volume of total DNA including both human and bacterial DNA from each of the extracts was 50μl to obtain the best possible concentration of TPA DNA.

#### PCR approach

We preliminarily produced a sequence of the HV1 region of mitochondrial DNA for each sample, as a control for the presence of DNA. Primers specific to the 23S rDNA region of the TPA genome were designed using Primer3web v4.1.0. Amplification was performed using the HGS Diamond Taq kit (Eurogentec) and the PCR Reagents Set kit (Agena Bioscience). PCR cycles were performed using a thermal cycler (Biometra and Veriti) comprising a first denaturation step at 95°C for 3 min, followed by 45 cycles including a denaturation at 94°C for 30 sec, a hybridisation at 56°C for 30 sec, an extension at 72°C for 1 min and a final extension step at 72°C for 7 min. We used an SS14 TPA reference genome (access number CP004011.1) for all designs and alignments.

#### 12 SNP multiplex

All samples in which the bacterium was detected were subjected to a multiplex PCR analysis of twelve SNP markers designed to distinguish between different strains of *T. p. pallidum* using MALDI-TOF mass spectrometry.

#### Multiplex primers

We targeted 2 variants specific to *T. p. endemicum*, 2 specific to *T. p. pertenue*, 2 specific to TPA, 2 for the SS14 strain of TPA, 2 for the Nichols strain and 2 for the CW59 strain (within Nichols, included to take advantage of the available space and provide more opportunities for the experiment to succeed).

#### Targeted enrichment and Illumina sequencing

A custom panel of 14216 80bp probes covering 99.85% of the TPA genome with no tiling was designed by the TWIST Bioscience supplier based on the SS14 strain reference genome (CP004011.1). Genomic DNA (gDNA, human + bacterial) from the FFPE samples was quantified using the Qubit™ 4 Starter Package (Invitrogen, USA). The concentrations obtained from the Qubit for our test samples were 0.161 ng/μl for sample 6709 and 1.32 ng/μl for sample 6715, both under the recommended 50ng of gDNA.

Library preparation was performed using 4.83ng of gDNA for sample 6709 and 46.2ng of gDNA for sample 6715. Sequencing libraries were prepared using Library Preparation with Enzymatic Fragmentation and the Twist Universal Adapter System (Twist Bioscience, San Francisco, USA) according to the manufacturer's instructions. The size range for each library was determined using the Agilent 2100 Bioanalyzer Instrument with the Agilent High Sensitivity DNA Kit.

The Twist Target Enrichment protocol generates target-enriched DNA libraries for sequencing on Illumina. We adjusted hybridisation temperature from 70°C to 62°C to optimise the reaction given fragment sizes (227-305 bp). The enriched library was quantified in the same way as previously described. After denaturation, the library was diluted to 12pM and as a control sample, PhiX DNA was added. Sequencing was performed using a standard Illumina flow cell (NGS) on the MiSeq (Illumina, San Diego, CA, USA) and the workflow was GenerateFASTQ.

#### Comparison of sequence Strasbourg_6715 to the phylogeny

We compared Strasbourg_6715 to the five closest samples in the phylogeny and analysed the distribution of SNPs across the genomes. Our results show that they are not strictly concentrated in one region but that certain regions are overrepresented, most notably the 23S ribosomal RNA, an outer membrane protein, TprI, TprJ and TprK.

### Quantification and statistical analysis

FastQ files generated by the Illumina MiSeq were treated using the EAGER pipeline v2.4.5.^34^ The quality of sequenced reads was estimated using FastQC V0.11.9^35^ and the adapters were removed using AdapterRemoval V2.3.2.^36^ Sequenced reads were mapped against a TPA strain SS14 (CP004011.1) using BWA v0.7.17.^37^ Duplicate reads were removed using markduplicates v2.26.0.^38^ Consensus sequences were generated using Samtools Consensus (Samtools v1.16.1^39^) under the ‘output all bases’ parameter and not showing insertions.

The complete sequence for the sample for patient 6715 was aligned against 269 published sequences of the three subspecies of human *T. pallidum* (*T. p. pallidum*, *T. p. pertenue*, *T. p. endemicum*). These published sequences were collected from the NCBI database and represent all available complete genomes for which dating and strain information was available [Table S2]. A prior alignment of these sequences was performed against the same TPA SS14 reference (CP004011.1) using MAFFT v7.505^40^ with a PAM of 1 and no iterative refinement. The sample from patient 6709 was excluded from these analyses.

The phylogeny was reconstructed using iqtree v2.0.3^41^ and 1000 bootstrap replications. We used TempEst v1.5.3^42^ and the original sampling dates to root the tree. The phylogenetic tree was visualised using the ape package^43^ for R.^44^ The characterization of contaminating sequences was performed using Kraken.^45^^,^^46^

## Supplemental information

Document S1. Figures S1‒S7

Table S1. Details of each case related to Table 1

Table S2. Metadata used for the phylogeny related to Figure 2
