# Supplementary material for: Evaluation of whole-genome enrichment and sequencing of T. pallidum from FFPE samples after 75 years
Source: iScience. 2023 Dec 8;27(1):108651. doi: 10.1016/j.isci.2023.108651 (PMC10753063; doi:10.1016/j.isci.2023.108651)
Supplement: Document S1. Figures S1‒S7 [file mmc1.pdf]

iScience, Volume *27*

Supplemental information

Evaluation of whole-genome enrichment and sequencing of *T. pallidum* from FFPE samples after 75 years

Vincent Zvenigorosky, Angéla Gonzalez, Gilles Veith, Tricia Close-Koenig, Catherine Cannet, Jean-Luc Fausser, Alexandre Wenger, Laurence Toutous-Trellu, Christine Keyser, and Christian Bonah

SUPPLEMENTARY INFORMATION

Evaluation of whole-genome enrichment and sequencing of *T. pallidum* from FFPE samples after 75 years

Zvenigorosky V.[1], Gonzalez A.[2], Veith G.[2], Close-Koenig T.[1], Cannet C.[2], Fausser J-L.[2], Wenger A.[3], Toutous-Trellu L.[4], Keyser C.[5], Bonah C.[1]

[1] SAGE Laboratory (CNRS UMR 7363, Strasbourg, France)

[2] Strasbourg Institute of Legal Medicine (France)

[3] Interfaculty Centre for Bioethics and Medical Humanities, University of Geneva (Switzerland)

[4] University Hospitals of Geneva (HUG, Switzerland)

[5] BABEL Laboratory (CNRS UMR 8045, Paris, France)

Contents

Molecular methods 2

Observed Spirochaete and contaminants 4

Comparison of sequence Strasbourg_6715 to the phylogeny 6

Molecular methods

Detection PCR primers (related to STAR Methods Planned progression of analyses for the proof-of-concept)

OLIGO	len	tm	gc%	sequence
**LEFT PRIMER**	22	58·62	50·00	TCATCCTTCTCCTTCACCCTAC
**RIGHT PRIMER**	22	59·32	50·00	TACTACCTACTTTCCCGCGAAG

Multiplex primers (related to STAR Methods Planned progression of analyses for the proof-of-concept)

SNP	Position in reference	First Primer	Second Primer
END (C/T)	266263	TGTTGATTTGGTGGTGTGGC	AAATGACTGCTGTTCGTGCC
END (C/T)	960288	TTTGTCGCGTCTCTGGAAGG	TATTTCCGTGGGAAGACGTG
PER (T/C)	598905	TTCTCGGGTGTCTTCCTCTG	TGAACCGACGGAAGAAGTAG
PER (C/T)	629622	CTGGAGAATTTTCTCCACGG	AGTCTACCGATGTCGATGTG
TPA (A/G)	718266	GCTAAAAGCTTTACGCGCTG	ATGTGAAGGTCAGGAACGTG
TPA (C/T)	734578	TTATCAGTACTCGCTCGAGG	TTCTTCCACCCTTGGAAGTC
SS14 (G/T)	199087	TGGGAACGATGTATCTTCGC	GCCCCTCAATACTGAAAATG
SS14 (C/T)	94196	GGAACTGCTAGCTGAGTATG	CTAGGAAGCTCTGGCTAAAG
NIC (C/A)	88128	TATGGTTGCGCATATGGTCC	GAACGAAACGCTGTGCATTC
NIC (C/A)	944644	TCATGTTCGCACTTCCTGCC	TCTTTGGACGGTGCGTTTAC
CW59 (G/A)	205961	TTAAACGCAGACCCCTTGAC	TAGAGCTGGTGGAAGAAGAG
CW59 (G/A)	540537	TGACCATCCGCGGTTTAATG	ATCGCGGACATGGATACTAC

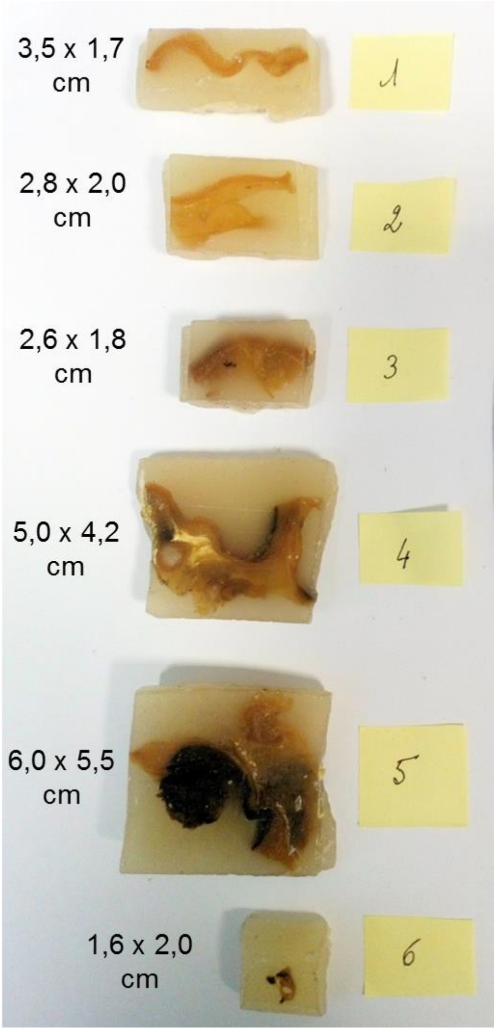

Figure S1 – FFPE blocks of varying sizes

(related to STAR Methods Planned progression of analyses for the proof-of-concept)

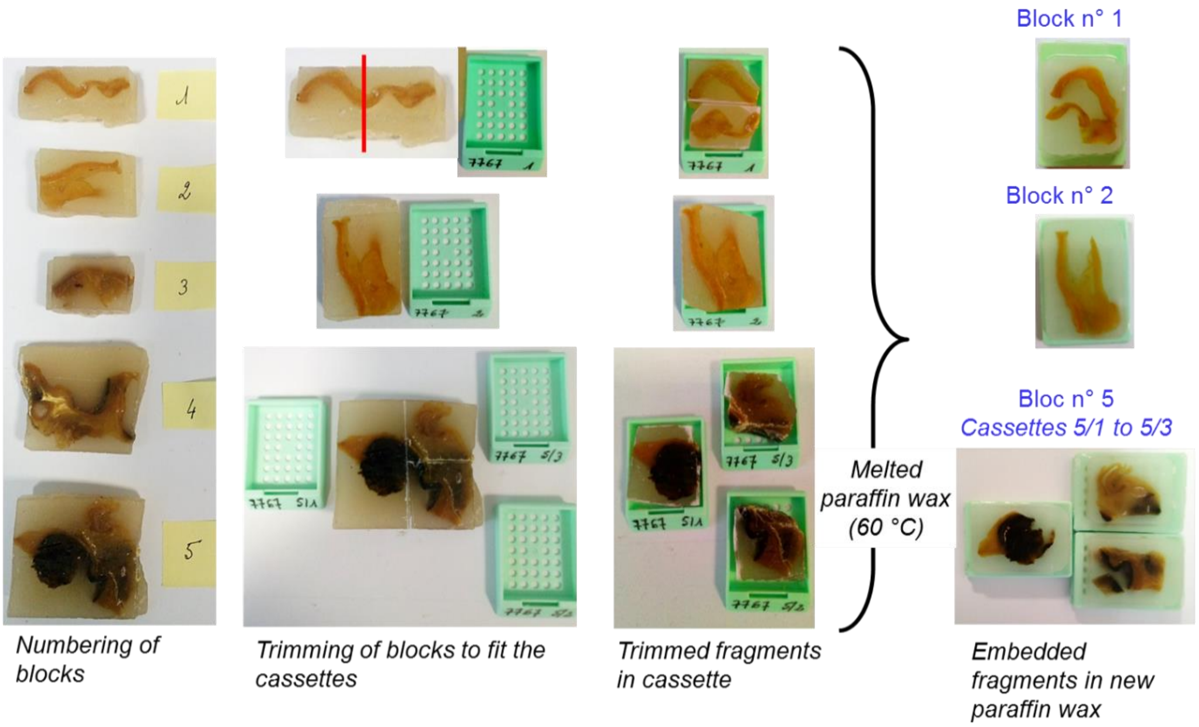

Figure S2 – Re-cutting and re-embedding process

(related to STAR Methods Planned progression of analyses for the proof-of-concept)

Observed Spirochaete and contaminants

The observed spirochaetes in case 6715 are presented in the main text of this study. The following figures present the positive control (infected rabbit testes), other positives and the contaminating fungi.

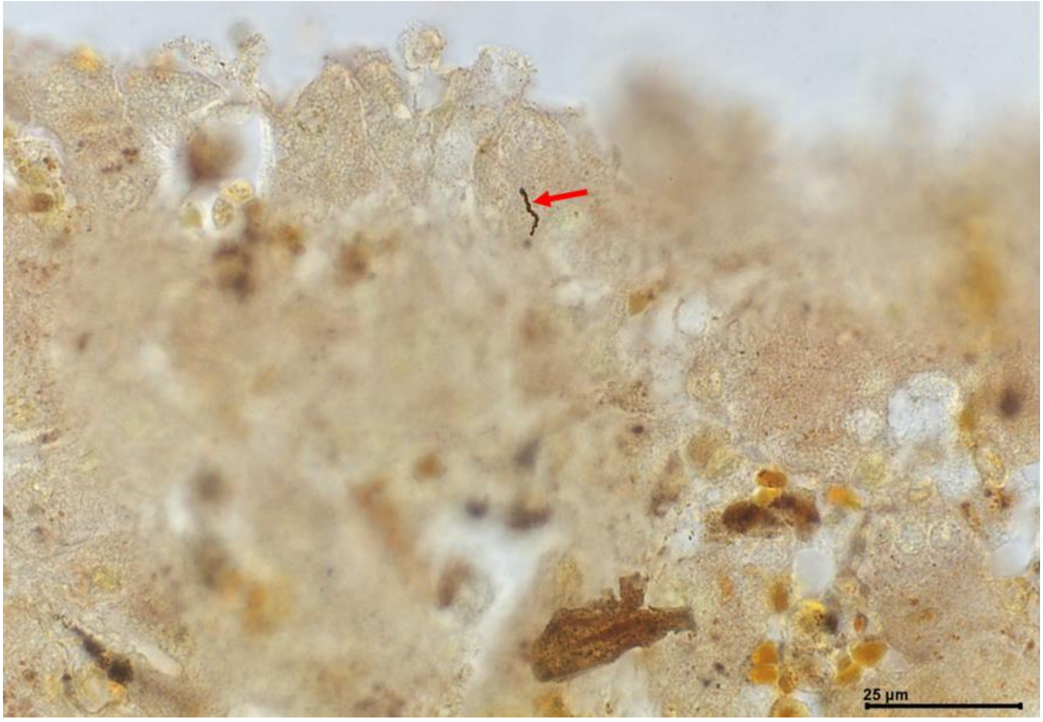

Figure S3 - Histological characterisation of case 6709 (related to Results: Histological positives)
Liver sample from a congenital syphilis case in 1947, fixed in Bouin; PCR positive

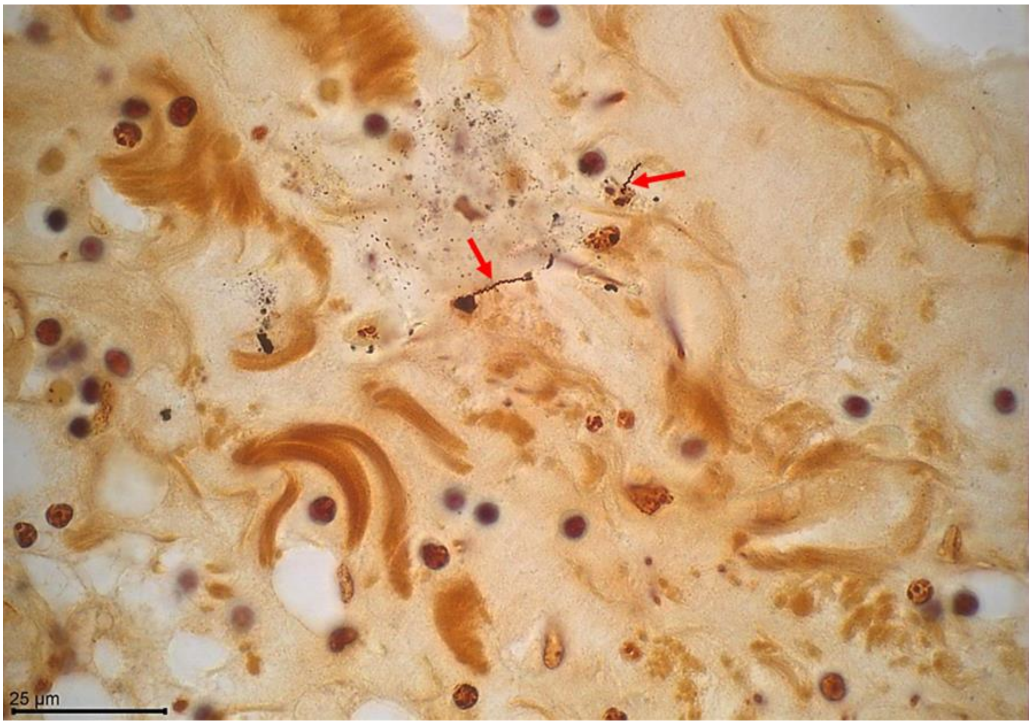

Figure S4 - Histological characterisation of case 8237 (related to Results: Histological positives)
Aorta sample from a syphilitic aortitis case in 1953, fixed in Bouin; PCR not tested

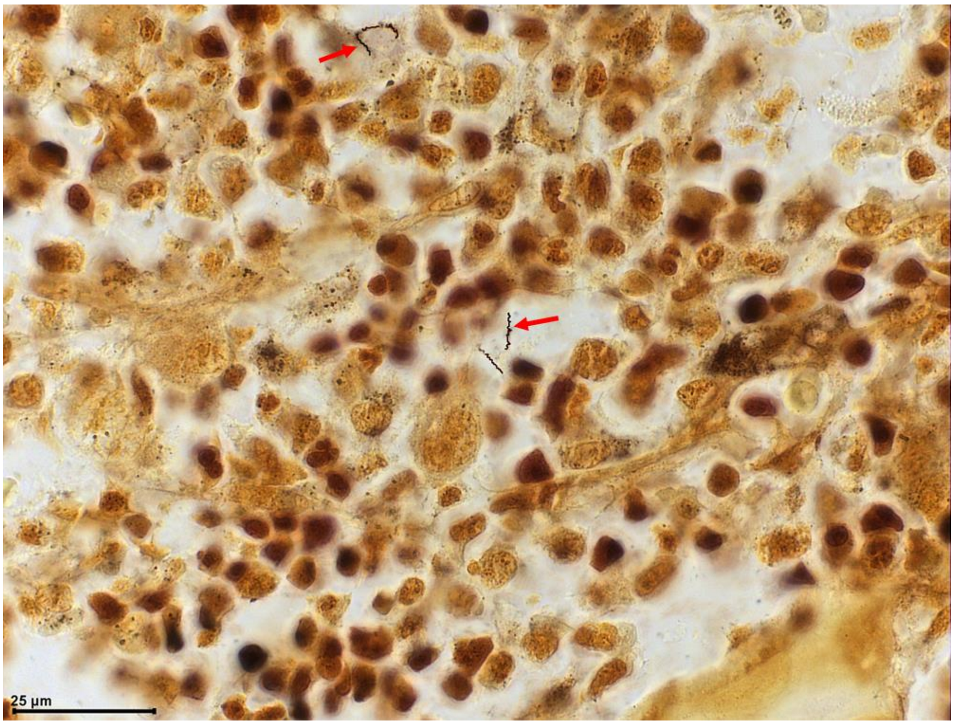

Figure S5 - Histological characterisation of case 12435 (related to Results: Histological positives)
Femur sample from a congenital syphilis case in 1960, fixed in Bouin; PCR negative

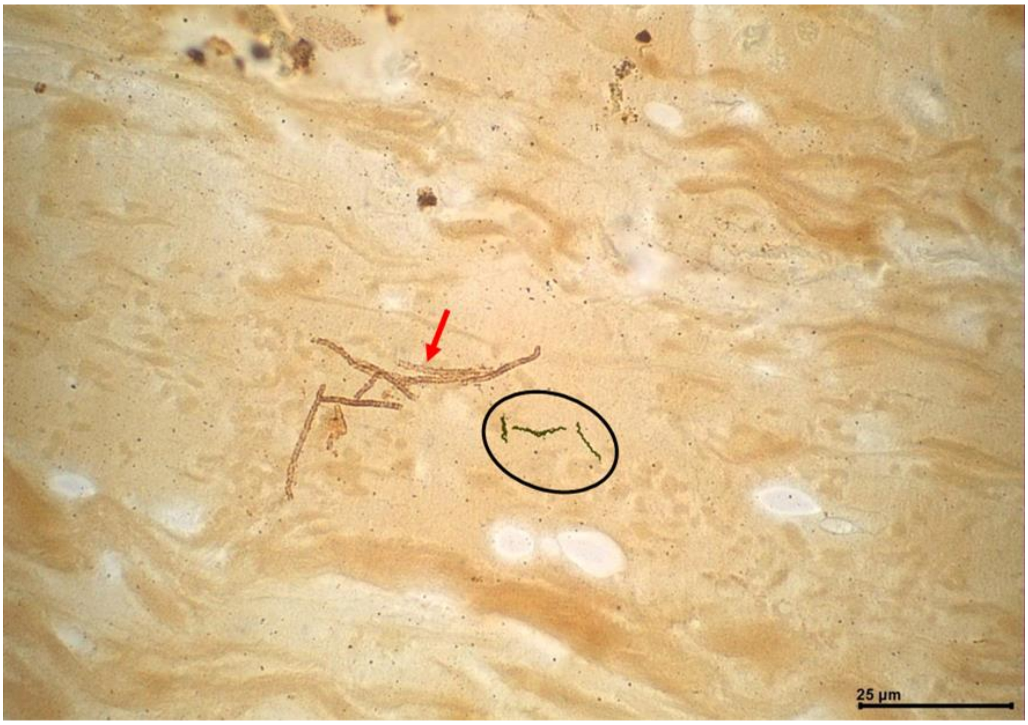

Figure S6 – Mould growing inside an FFPE block (related to Results: Histological positives)
Black circle indicates spirochaete bacteria; red arrow indicates contaminating mould

Comparison of sequence Strasbourg_6715 to the phylogeny

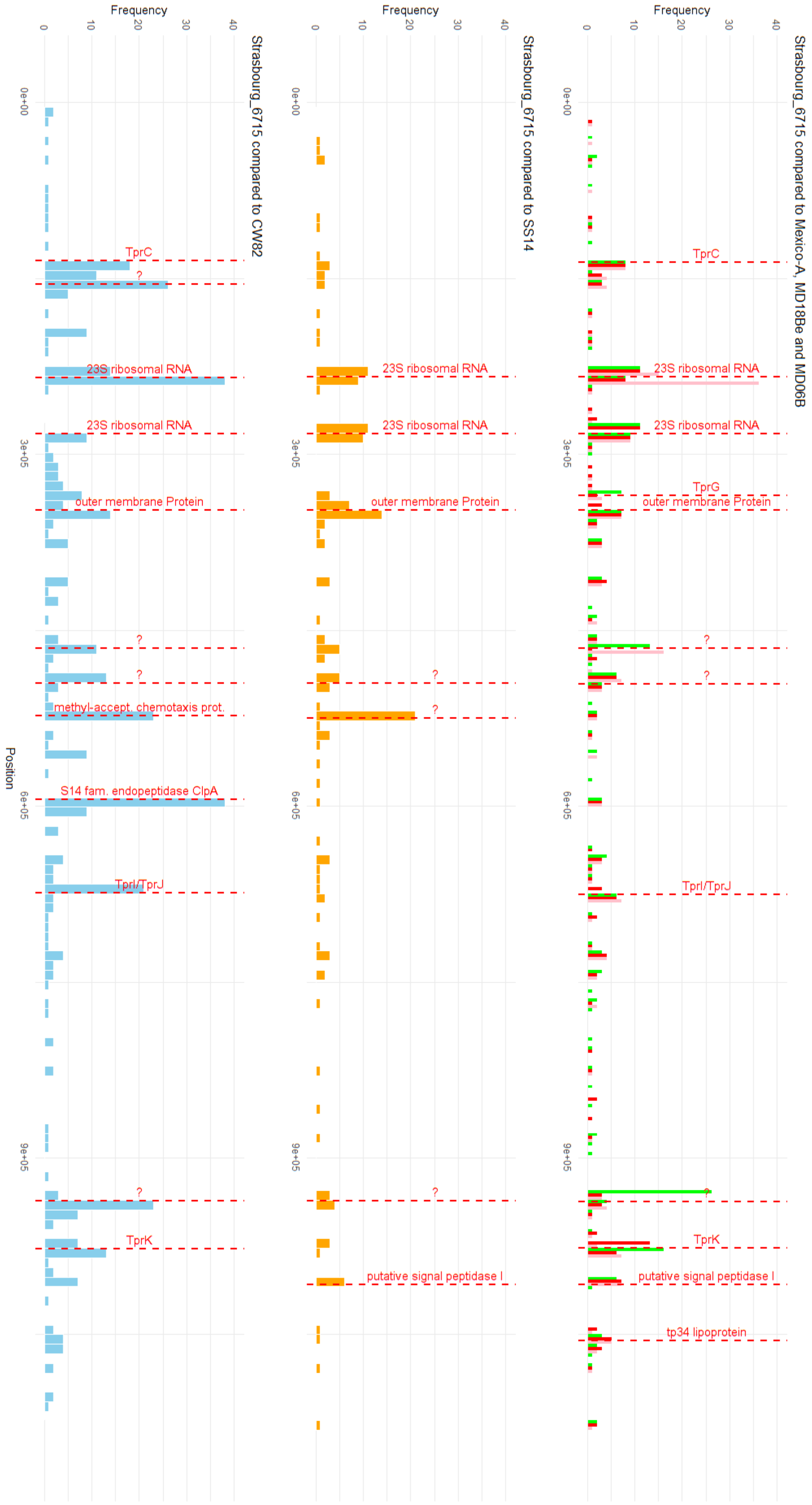

<u>Figure S7 – Distribution of SNP of Strasbourg_6715 compared to closely related sequences (related to Results: Phylogeny)</u>

Comparisons:
blue: CW82;
orange: SS14;
green: MD06B;
red: MD18Be;
pink: Mexico-A;

red dotted lines: genes of interest in high-variability regions
?: hypothetical proteins
